# Neutrophil extracellular traps in rheumatoid arthritis: biomarkers, drivers, and emerging therapeutic targets

**DOI:** 10.1093/cei/uxag011

**Published:** 2026-02-18

**Authors:** Sangeeta Kumari, Katerina Pardali, Eric Meldrum, Christian Lood, Maarten Kraan

**Affiliations:** Research and Development, Citryll BV, Pivot Park, Oss, The Netherlands; Research and Development, Citryll BV, Pivot Park, Oss, The Netherlands; Research and Development, Citryll BV, Pivot Park, Oss, The Netherlands; Division of Rheumatology, Department of Medicine, University of Washington, Seattle, WA, USA; Research and Development, Citryll BV, Pivot Park, Oss, The Netherlands

**Keywords:** neutrophil extracellular traps, NETosis, citrullinated histones, rheumatoid arthritis, biomarkers

## Abstract

Neutrophil extracellular traps (NETs) are web-like structures composed of DNA, histones, and granule proteins released by activated neutrophils. While originally characterized as part of the innate immune response, NETs are now recognized as contributors to the pathogenesis of immune-mediated inflammatory diseases, including rheumatoid arthritis (RA). This review summarizes current clinical evidence linking NETs to RA, with a focus on their utility as biomarkers for disease activity and treatment response and their potential mechanistic role in disease progression. Elevated levels of NET components, such as myeloperoxidase–DNA complexes, citrullinated histones, and calprotectin, have been reported in RA and correlate with inflammatory markers and clinical disease activity scores. Treatment with biological disease-modifying anti-rheumatic drugs, including tumour necrosis factor alpha and interleukin-6 inhibitors, reduces NET markers, whereas persistent NET formation is associated with poor response. NETs also promote pathogenic processes, including anti-citrullinated protein antibody formation, Th17 activation, and osteoclastogenesis. Although no therapies currently target NET formation directly, preclinical studies using PAD4 inhibitors and antibodies against citrullinated histones show promising effects. Standardizing NET biomarkers and conducting longitudinal studies will be essential for clinical translation. Overall, NETs represent both a biomarker and a mechanistic driver in RA, offering a novel opportunity for therapeutic intervention.

## Teaser

Neutrophil extracellular traps (NETs) play an important role in the development of rheumatoid arthritis (RA). Clinical evidence shows that components of NETs are linked to disease activity in RA, treatment response, and important disease processes such as autoantibody formation and bone destruction. Altogether, NETs have the potential to serve as both a useful biomarker and a therapeutic target for RA.

## Graphical Abstract

**Graphical Abstract:**
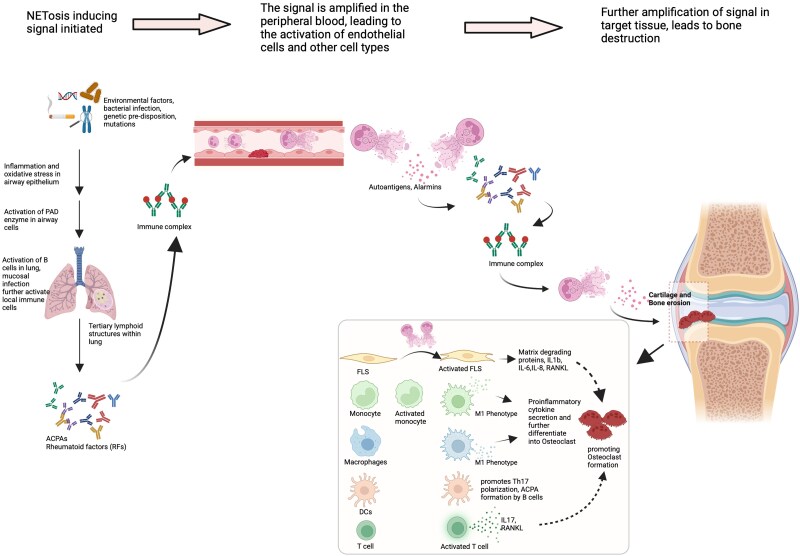
NETosis can be triggered by various environmental factors in genetically or immunologically susceptible individuals. These initial triggers lead to the activation of PAD enzymes, mucosal cells, and neutrophils, ultimately resulting in the formation of neutrophil extracellular traps (NETs). As a rich source of autoantigens, NETs stimulate local B cells to produce anti-citrullinated protein antibodies (ACPAs). The ACPA pool then expands and enters systemic circulation, forming immune complexes. Immune complexes along with proinflammatory mediators released during NET formation activate various circulating immune cells, thereby further amplifying the NETosis response. In target tissues, NETs and their components activate a range of cell types, including fibroblast-like synoviocytes (FLSs), monocytes, macrophages, dendritic cells (DCs), T cells, and B cells. This cascade of interactions creates a highly inflammatory milieu that contributes to cartilage degradation and bone erosion [Created in BioRender. Kumari, S. (2026) https://BioRender.com/sx5nxrq].

## Introduction

Neutrophils are the most abundant cells of the innate immune system, contributing to host defence. Neutrophils are among the first immune cells recruited to sites of inflammation and, when activated under appropriate conditions, orchestrate both the progression and resolution of inflammation by regulating the functions of other immune cells [1, 2]. Their contribution to host defence is mediated through multiple mechanisms, including the production of reactive oxygen species (ROS), degranulation, phagocytosis, and the formation of neutrophil extracellular traps (NETs) [1, 2]. The formation of NETs occurs by an ordered series of events during which the nuclear and granule membranes dissolve and chromatin decondenses. This process generates the force that results in cell rupture and the release of extracellular, web-like chromatin structures termed NETs, which are decorated with cytoplasmic and granular contents. NETs were initially observed in the context of neutrophil responses to bacteria and are cytotoxic, proinflammatory, and immune cell activating and influence cell fate determination and differentiation [2, 3]. Increasing evidence indicates that excessive formation and impaired clearance of NETs in the absence of infectious agents are associated with the pathogenesis of numerous immune-mediated inflammatory diseases, including rheumatoid arthritis (RA).

In RA, the presence of large numbers of activated neutrophils, cell-free DNA, and granule proteins in the synovial compartment is suggested to play a pathogenic role in disease [4].

However, uncertainties remain concerning which NET markers most accurately reflect disease state, how serum NET markers change following response to standard-of-care treatment, and whether NET markers can be used to describe disease activity and progression or for predicting treatment response. Ultimately, it remains to be established whether NET-targeting approaches effective in preclinical models of arthritis can achieve similar anti-inflammatory outcomes in RA patients. This article aims to compile the NET-associated clinical data available in RA to address these uncertainties.

## Materials and methods

We performed a targeted literature search to summarize the evidence from published studies involving samples from human subjects on the role of NETs and the potential of targeting NET formation and NET remnants by therapeutic interventions. The search was performed in three different databases: PubMed, Cochrane, and Clinical trial.gov. To facilitate the translatability of the concept of targeting NETs in the clinical landscape, the search strategy focused on studies involving human subjects with animal studies excluded. Search criteria included: ‘NETosis and RA’, ‘NET markers in RA’, ‘neutrophil extracellular traps and RA’, ‘citrullinated histones in RA’, ‘nucleosome and myeloperoxidase (MPO) in RA’, ‘disease modifying anti-rheumatic drugs (DMARDs) and NET levels’, ‘NETosis and RA and disease activity’, and ‘NETosis marker and RA’.

The screening included all relevant articles irrespective of year of publication. To ensure relevant articles were included, searches were performed twice by the same person. After listing, the articles were assessed and numbered according to the level of confidence based on a predefined set of criteria. These criteria included the perspective of the study, sample size, sample type (randomized vs. controlled), attached background clinical information such as disease activity, treatment status, and the specific assays used in the study. References from retrieved and selected articles were also scanned for relevant publications. Once articles were ranked, they were further categorized to select for the considerations raised in this review article.

## Results

### Numbers of articles screened and structure of this review

**Figure 1:**
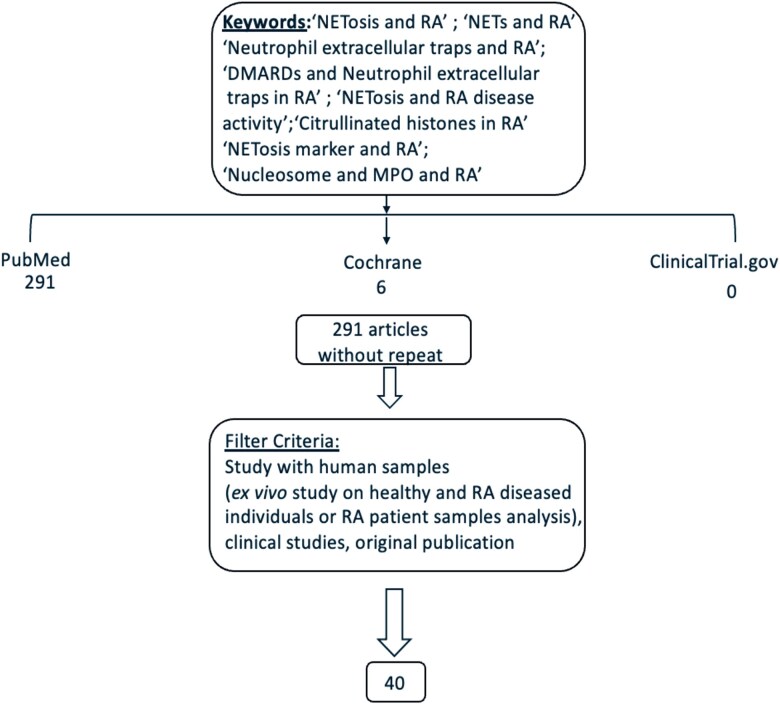
Summary of the literature search [Created in BioRender. Kumari, S. (2026) https://BioRender.com/92p23ae].

Two hundred ninety-one peer review manuscripts were identified, and of these, 251 were excluded based on the predefined exclusion criteria (Fig. 1). All 40 selected articles describe studies with samples derived from either healthy volunteers or RA patients.

Based on a review of the literature, we have summarized our findings as follows: the characteristics of neutrophils in RA, the significance of NETs and their components in RA disease progression and monitoring disease activity, and finally, a discussion on the potential of therapeutically targeting NETs.

### RA neutrophils are prone to form NETs

Most articles screened agree that neutrophils from RA patients display an altered state compared with those of healthy volunteers. Neutrophils isolated from both peripheral blood and synovial fluid of RA patients have increased propensity to form NETs compared with neutrophils from healthy controls and osteoarthritis (OA) patients [5, 6]. Neutrophils from RA patients form NETs spontaneously in culture without the need of additional stimuli. Furthermore, under both spontaneous and stimulated conditions, neutrophils from RA patients generate NETs more rapidly and to a greater extent than healthy neutrophils [6, 7].

With histologic examination, NETing neutrophils can be detected infiltrating the synovial tissue, rheumatoid nodules and skin of RA patients [6]. Correspondingly, RA synovial fluid neutrophils exhibit delayed apoptosis, increased chemokine expression, elevated ROS production and activation of AKT, RAF1, and SRC-regulated signalling cascades, which collectively lead to NET formation [8]. These observations suggest a primed state for RA neutrophils to form NETs in joints and peripheral blood. Apart from the propensity of RA neutrophils to form NETs, the IgG pool from anti-citrullinated protein antibody (ACPA)-positive RA serum can further stimulate neutrophils to form NETs in a concentration-dependent manner [9].

In RA and other autoimmune diseases like systemic lupus erythematosus (SLE), the presence of a neutrophil subset known as low-density granulocytes (LDGs) is found in peripheral blood [10]. Named for their presence in the PBMC layer during density gradient centrifugation, LDGs display traits of both mature (cell surface markers) and immature neutrophils (e.g. expression of CDK2, CDK4, and CDK6 transcripts) [11]. In SLE, LDGs show enhanced NET formation. Similarly, RA LDGs have altered transcriptomes with elevated granule protein and cell cycle gene expression, along with increased survival. One study reports that, unlike in SLE, RA LDGs do not exhibit increased NET formation; however, further research is needed to clarify their role in RA [12]. Moreover, proteomic analyses of RA synovial fluid reveal abundant neutrophil-derived inflammatory proteins and cell-free DNA, which, together with hypoxia, autoantibodies, and anti-apoptotic cytokines, promote neutrophil recruitment, activation, and survival [1, 2, 13]. Taken together, the available evidence underscores that neutrophils in RA exhibit unique phenotypic and functional properties that critically contribute to the immunopathology of the disease.

### Proteins associated with NET formation are elevated in RA

Enhanced levels of NET components, including cell-free DNA, cell-free nucleosomes, neutrophil elastase (NE), MPO, and MPO–cell-free DNA complex, have been detected in the serum and plasma of RA patients [5, 7, 14].

The above markers, together with citrullinated histones, calprotectin, calgizzarin, and NE–DNA complex, are elevated in patient plasma and serum samples (Table 1) and are indicative of NET formation. Furthermore, associations between the level of serum NET remnants (specifically MPO–DNA and NE–DNA complexes) and ACPA titres, as well as sputum NET level (measured by DNA–MPO and DNA–NE) and anti-CCP levels, have been described in RA [16, 18, 30].

**Table 1:** Markers evaluated in different studies quantifying NET-associated factors in RA patient samples.

Marker	References	Number of samples in the study	Matrix	Association with DAS28	Methodology of NET measurement
MPO–DNA	Navratilova *et al*. [15]	30	Synovial fluid	Yes	ELISA
	Navratilova *et al*. [15]	8	Synovial tissue	No	IF
	Bach *et al*. [5]	41	Plasma	Yes	ELISA
	Wang *et al*. [16]	74	Serum	No	ELISA
	Li *et al*. [17]	86	Serum	Yes	ELISA
	Wu *et al*. [18]	51	Serum and plasma	N/A	ELISA
	Chowdhury *et al*. [7]	32	Serum	No	ELISA
	Ye *et al*. [19]	25	Serum	Yes	ELISA
	Ye *et al*. [19]	5	Synovial tissue	Yes	IF
NE	Frade-Sosa *et al.* [20]	114	Plasma	No	ELISA
	Ruiz-Limón *et al*. [21]	106	Serum	Yes	ELISA
	Navratilova *et al*. [15]	60	Serum	No	ELISA
	Wu *et al*. [18]	51	Serum	N/A	ELISA
	Chowdhury *et al*. [7]	32	Serum	No	ELISA
	Ye *et al*. [19]	25	Serum	Yes	ELISA
	Ye *et al*. [19]	5	Synovial tissue	Yes	IF
Citrullinated histone epitopes	Navratilova *et al*. [15]	30	Synovial fluid	Yes	ELISA
	Peng *et al*. [22]	151	Serum	Yes	ELISA
	van der Linden *et al*. [23]	6	Synovial tissue	N/A	IHC
Histone–DNA	Frade-Sosa *et al.* [20]	114	Plasma	No	ELISA
NE–DNA	Kuley *et al*. [24]	271	Plasma	Yes	ELISA
	Frade-Sosa *et al.* [20]	114	Plasma	No	ELISA
Calprotectin	García-Arias *et al*. [25]	60	Serum	Yes	ELISA
	Bach *et al*. [5]	441	Plasma	Yes	ELISA
	Sejersen *et al*. [26]	76	Serum	Yes	ELISA
	Gernert *et al*. [27]	114	Serum	Yes	ELISA
	Jarlborg *et al*. [28]	969	Serum	Yes	ELISA
	Frade-Sosa *et al.* [20]	114	Plasma	Yes	ELISA
	Kuley *et al*. [24]	271	Plasma	Yes	ELISA
Calprotectin–DNA	Fagerhol *et al*. [29]	8	Serum and plasma	N/A	ELISA
Nucleosome/DNA	Ruiz-Limón *et al*. [21]	106	Plasma	Yes	ELISA
	Chowdhury *et al*. [7]	32	Serum and plasma	No	ELISA
PAD4 activity	Navratilova *et al*. [15]	30	Synovial fluid	Yes	ELISA

While the aforementioned markers are widely used to describe elevation of NETs, it is of relevance to note that a reliable and universally accepted marker of NETs remains to be established. Investigators generally combine a variable panel of multiple markers for composite findings indicative of NETs. Threshold levels of these markers for differentiating individuals with active disease from healthy have not been determined, and consequently, their future utility in clinical practice remains to be defined.

Table 1 summarizes markers evaluated in different studies quantifying NET-associated factors in RA patient samples.

In addition to these commonly assessed NET markers, novel markers have been suggested to be linked to NETs. B-cell-associated cytokine interleukin (IL)-40 and the S100 family member protein Calgizzarin (S100A11) are two such NET-associated markers described in recent publications [15, 31]. IL-40 colocalization is observed with MPO in synovial tissue and is proposed to be formed by NET-forming neutrophils [15]. IL-40 has also been shown to be increased in the serum, synovial fluid, and synovial tissue samples of RA patients. Similarly, Calgizzarin levels are increased in synovial fluid samples of RA patients along with other markers of NET formation: CitH3, MPO, and PAD enzyme activity [15].

### Molecular and cellular factors underlying enhanced NET formation in RA

NET formation in RA is driven by a combination of factors, including genetic predisposition, environmental factors, and immune activation. Key inducers of NET formation include inflammatory cytokines, autoantibodies, and, more recently, *N*-formyl methionine peptides derived from damaged tissues and/or mitochondria [6, 7, 32]. Among genetic contributors, mutations in the PADI4 gene, variants such as PTPN22 and HLA-DRB1 shared epitope alleles, have been implicated in enhanced NET formation [33–35]. Several single nucleotide polymorphisms (SNPs) within PADI4 are associated with increased RA susceptibility by promoting higher expression levels or greater enzymatic stability of PAD4, thereby facilitating histone citrullination and NET formation [33]. Another example is the PTPN22 gene, which normally acts as a negative regulator of PAD4 enzymatic activity and protein citrullination but loses its inhibitory function in carriers of the C1858T polymorphism. This SNP causes a substitution of arginine (R620) with tryptophan (W620), impairing PTPN22’s function and resulting in increased citrullination, ACPA production, and NET formation [34]. Similarly, the shared epitope region of HLA-DRB1 gene is a significant genetic risk factor for the development of RA. It is believed that RA-associated HLA-DRB alleles present citrullinated peptides to T cells that help ACPA-producing B cells [36]. In genetically predisposed individuals, environmental exposures such as mucosal infections or cigarette smoke may serve as initiating triggers that activate PAD4 and neutrophils, thereby setting off the pathogenic cycle of NET formation to perpetuate inflammatory responses in RA.

### NETs contribute to an enhanced citrullinated auto-epitope pool in RA

Autoantibodies identified in the blood of RA patients, such as rheumatoid factor (RF) antibodies targeting the Fc portion of IgGs and ACPAs, can be detected years prior to the onset of disease and are commonly used in diagnostic tests [37]. Opinions regarding the origin of ACPAs vary, with genetic factors (HLA-DRB1 allele, PADI4 SNPs, and mutation like PTPN22) together with environmental factors (smoking, dust, and bacterial pathogen) contributing to initial events that lead to activation of PAD enzymes and generation of citrullinated epitopes [38]. These initial events are further amplified by the response of activated neutrophils that undergo NETs formation, thereby increasing the citrullinated auto-epitope pool and instigating ACPA generation. Two such studies underscore the significance of local inflammation with NET formation in promoting anti-CCP production and the early development of RA [30, 39].

Moreover, proteomic analysis of RA serum revealed that NETs are decorated with citrullinated vimentin and citrullinated α-enolase, the two most frequently observed autoantigens in RA [6, 40]. However, the sera of RA patients react diversely with different citrullinated proteins, not only against histones and intracellular antigens but also against extracellular proteins such as fibrinogen [41]. This could be explained by the fact that during NET formation, not only are citrullinated epitopes expelled, but PAD enzymes are also released into the extracellular environment. These extracellular PADs can citrullinate proteins that are normally not modified under healthy conditions. As a result, these newly citrullinated proteins are recognized as foreign by the immune system, contributing to the expansion of the ACPA repertoire.

While NETs are one source of autoantigens sustaining ACPA production, they also induce the expansion and differentiation of ACPA-producing B cells. This is proposed to take place in regional lymph nodes and ectopic germinal centres where T cells, B cells, and plasma cells collectively contribute to NET uptake and processing, leading to a further increase in the ACPA pool. This, in turn, promotes the initiation and/or progression of joint inflammation [42].

### Elevated NET levels associate with cartilage and bone loss in RA patients

The presence of abundant neutrophils and NETs in inflamed synovial joints has prompted investigation into their potential contribution to joint tissue damage in RA. NETs are decorated with cytotoxic citrullinated and carbamylated histones as well as neutrophil granule enzymes, which together can degrade extracellular matrix components and accelerate bone and cartilage destruction. Consistent with this, NET levels are elevated in synovial fluid from RA patients with active bone erosion and positively correlate with the synovial fluid RANKL/OPG ratio, a key indicator of the balance between bone resorption and formation [43, 44].

NET-associated NE contributes directly to cartilage damage by mediating degradation of aggrecan, the most abundant structural proteoglycan in human articular cartilage [45]. Carmona-Rivera *et al*. demonstrated that incubation of aggrecan with NETs resulted in significant proteoglycan degradation, which was markedly attenuated in the presence of the NE inhibitor sivelestat, highlighting a direct role for NET-bound proteases in cartilage erosion.

In addition to their direct tissue-destructive capacity, NETs serve as a source of carbamylated proteins, against which RA patients frequently develop autoantibodies [44, 46]. O’Neil *et al*. showed that autoantibodies targeting carbamylated histones correlate with the extent of bone erosion in RA. Elevated levels of carbamylated NET (cNET) proteins in plasma and synovial fluid of RA patients, but not OA controls, were further associated with increased systemic markers of bone turnover and inflammation [44]. Mechanistically, NETs, and particularly cNETs, were shown to activate tissue macrophages to release proinflammatory cytokines and to stimulate fibroblast-like synoviocytes to produce RANKL, thereby directly promoting osteoclast differentiation and inflammatory bone loss.

### NETs contribute to cardiovascular disease development in RA

NETs are enriched in factors like dsDNA, nucleosomes, and citrullinated histone, which are known to exert cytotoxic and prothrombotic effects across a range of inflammatory and cardiovascular diseases, including RA. NET-associated proteins, including NE and MPO, further amplify inflammatory and coagulation pathways [47–49].

The presence of NET markers such as citrullinated histone H3 (CitH3), extracellular DNA, NE, and MPO has been consistently demonstrated in human thrombotic samples, including arterial thrombi, cerebral thrombi, and pulmonary emboli, supporting a direct role for NETs in thrombus formation [50–52]. In addition, circulating NET components have been linked to systemic thromboinflammatory activity. In a prospective, observational cross-sectional study of 282 individuals with suspected coronary atherosclerosis, plasma levels of NET-derived factors, including dsDNA, nucleosomes, citrullinated histone H4, and MPO–DNA complexes, were positively associated with thrombin generation and were significantly elevated in patients with severe coronary atherosclerosis or extensively calcified coronary arteries [48]. These findings support a link between NET burden, coagulation activation, and vascular pathology in humans.

Mechanistically, NET-associated DNA and histones provide a structural scaffold that promotes platelet adhesion and activation while also facilitating activation of the intrinsic coagulation cascade. Importantly, NETs have been shown to carry or induce the expression of tissue factor, thereby linking innate immune activation directly to thrombin generation [53]. In the context of RA, these mechanisms are of particular relevance given the well-established increase in cardiovascular morbidity and mortality observed in this patient population. RA patients experience a substantially elevated risk of cardiovascular disease, which cannot be fully explained by traditional risk factors alone [54]. In a meta-analysis encompassing 111 758 RA patients, RA was associated with an ∼50% increased risk of cardiovascular mortality compared with the general population [55]. Chronic systemic inflammation, coupled with dysregulated neutrophil activation and NET formation, may therefore represent an important mechanistic link between RA and heightened thromboinflammatory cardiovascular risk.

### Correlation of NET markers to RA disease activity

Numerous reports demonstrate the recruitment of neutrophils into the inflamed joint, and the presence of NET markers in blood, synovial fluid, and tissue contributes to a circumstantial case for the participation of proinflammatory NETs in disease pathogenesis.

Quantification of NET-derived products in the plasma of RA patients and healthy controls demonstrates that both cell-free DNA and NE significantly correlate with clinical parameters, as measured by disease activity score counting 28 joint (DAS28) and acute phase reactants like erythrocyte sedimentation rate (ESR) and C-reactive protein (CRP) [14]. Elevated serum levels of the NET marker citrullinated histone H3 (CitH3) have also been shown to correlate significantly with increasing disease activity [22].

Significantly elevated levels of calprotectin in plasma from RA patients have been shown to correlate with both increasing Clinical Disease Activity Index and CRP [5, 25, 27, 28]. Calprotectin, a heterodimer composed of two different protein subunits, S100A8 and S100A9, is primarily expressed in neutrophils but is also found in monocytes, macrophages, and other cells. It is a biomarker associated with neutrophil activation, NET formation and disease activity [56]. A study on serum samples from 76 RA patients showed a significant correlation of serum calprotectin with disease activity and inflammatory markers in ACPA-positive RA [26]. Furthermore, serum calprotectin has been proposed as a marker for disease activity in response to biological DMARDs in a cross-sectional study of 33 RA patients receiving the anti-IL-6 receptor antibody tocilizumab (TCZ) [57].

However, conflicting data does exist. For example, in patients with varying disease activity receiving different treatment regimes, the relationship between plasma NET markers, calprotectin, and clinical and ultrasonographic disease activity was examined, with no association observed between plasma NET markers and inflammatory disease status [20]. In fact, this study by Frade-Sosa *et al*. raises questions about the utility of NET remnants in peripheral circulation as biomarkers for inflammatory activity. Whether calprotectin is indicative of neutrophil activation in RA or if it reflects an overall inflammatory status with contribution from several immune and structural cell types remains to be established. Similarly, some studies have reported negative findings, showing no correlation between other NET markers and disease activity. For instance, Wang *et al*. observed that although serum levels of MPO–DNA complexes were significantly elevated in RA patients compared with healthy controls, receiver operating characteristic analysis demonstrated low sensitivity and specificity in the overall RA cohort, with differing sensitivity and specificity between ACPA-positive and ACPA-negative patients. [16]. It is worth noting that although most studies report elevated NETs markers in RA compared with healthy volunteers, several of them demonstrate only weak or moderate correlations, or identify associations limited to specific patient subgroups [7].

### Effect of biological DMARD treatment on levels of NET markers

Successful treatment with biological disease-modifying drugs (bDMARDs) has been shown to reduce serum and plasma markers of NETs and neutrophil activation. A longitudinal study of 20 RA patients receiving weekly TCZ, treatment for the duration of 6 months reported significantly reduced serum cell-free DNA [21]. Furthermore, after TCZ treatment, patient neutrophils showed reduced expression of NE and MPO and reduced capacity to form NETs. In a later report, the same group evaluated an independent cohort of 75 RA patients for the effect of 6-month treatment with either infliximab (IFX), an anti tumour necrosis factor (TNF) antibody, or TCZ [14]. Serum markers of NETs, such as cell-free nucleosomes and elastase, were decreased in RA patients after treatment with IFX or TCZ. In another report, patients on various anti-TNF therapies were assessed for serum NET markers (NE and cell-free nucleosomes), oxidative stress markers, and proinflammatory cytokines. Those with poor clinical response had markedly higher levels of these markers compared with patients who responded well to treatment, pointing to increased activation of neutrophils [58]. Increased serum MPO–DNA levels have also been proposed as a marker of RA disease activity. Longitudinal serum samples from 86 RA patients treated with methotrexate and the Etanercept (anti-TNFα) biosimilar Yisaipu were studied for levels of serum MPO–DNA complex. A decrease in serum MPO–DNA levels after 4 weeks of treatment was shown to correlate with improved disease activity parameters such as CRP, ESR, SJC (swollen joint count), TJC (total joint count), and DAS28 [17].

### Technical challenges with NET assessment

The field of NET formation has evolved significantly over the last two decades, and so have the assays used to measure NETs. However, it is important to note that a lack of consensus on sample processing and assay formats for NET analysis has resulted in discrepant findings across the literature. Initial findings establishing NETs *in vitro*, as well as *in situ*, primarily relied on microscopic identification of extracellular DNA, often in a web-like structure, coated with neutrophil cytosolic components such as MPO or NE [3]. Electron and fluorescence microscopy remain the gold standard for identifying NETs *in vitro*, typically combining DNA dyes with neutrophil markers, such as MPO, NE, or CitH3. For measurement of NETs in patient samples, however, there is more discrepancy, both regarding what to measure and how to measure them. Regarding the ‘what to measure’ several manuscripts unfortunately to this day report on ‘NETs’ or ‘NET components’, while measuring generic markers of cell death, such as cell-free DNA, that could be derived from any cellular source, and/or neutrophil soluble markers, such as levels of NE or MPO. Although indicative of neutrophil activation, those markers alone are insufficient to reflect NET formation without evidence of DNA complexing. Instead, to achieve a high confidence in the measure, it is appropriate to measure either NE, MPO, or CitH3 in complex with DNA. Although enzyme-linked immunosorbent assay (ELISA)-based assays are generally considered the gold standard for NET detection, alternative approaches such as flow cytometry and imaging of blood smears have also been proposed [59, 60]. Although NE–DNA, MPO–DNA, and citH3–DNA are all representative of NET formation, few studies have evaluated them consistently to determine whether one or the other NET complex would be more suitable for use in a clinical use, with correlation studies showing only moderate correlation between the various markers [61].

An additional challenge with NET assays, in particular in autoimmune conditions, is the presence of autoantibodies targeting the autoantigens utilized by the detection assay, e.g. MPO, NE, citH3, and DNA. Anti-NET IgG is highly prevalent in most autoimmune conditions and is shown to affect the NETs degradation through sterically preventing DNase activity [62]. This is not least important in diseases such as SLE (autoantibodies towards chromatin, DNA, and histones), vasculopathies (autoantibodies towards neutrophil granular proteins), and RA (autoantibodies towards citrullinated peptides, including histone H3) [6, 63, 64].

Importantly, NET formation represents only one of several programmed necrotic death pathways in neutrophils, which also include pyroptosis, ferroptosis, and necroptosis in various inflammatory settings [65, 66]. Which of these cell death processes is dominating in the inflammatory joint, or elsewhere, has not been carefully studied. Further, it has not been carefully determined whether either of those other cell death processes, similarly to NET formation, can result in the release of MPO–DNA, NE–DNA, and/or citH3–DNA complexes. However, prior work using nigericin, a common agonist for induction of pyroptosis, demonstrated formation of ‘NET-like’ structures staining for DNA and NE [67, 68]. Thus, one needs to be careful in interpreting data on soluble ‘NETs’ as they may very well be derived through mechanisms other than NET formation.

Finally, one needs not only to consider what molecule to measure and how but also the biomaterial itself. It is important to note that neutrophils undergo activation upon coagulation, with serum levels not reflecting the true physiological levels of NETs experienced by patients but rather the propensity of neutrophils to undergo NET formation upon excessive clotting. While serum levels of NETs could still provide clinical implications, including in RA, plasma samples are preferable to determine physiological NET levels [5, 7, 14, 69]. However, also for plasma samples, the lack of standardized protocol for sample processing, including time to processing, centrifugation steps, and use of anticoagulant, hampers comparison between various publications, as well as collaborative studies across sites.

Overall, there is a need to establish technical guidelines, based on scientifically rigorous data, to define gold standard protocols for sample processing and NETs measurement to advance the field, and implement NET markers in the clinical realm.

## Discussion

NETs are increasingly recognized as potent contributors to both inflammation and tissue damage in RA. While NET formation is a critical mechanism of acute host defence, it becomes chronically activated in autoimmune settings like RA. NET formation has emerged as a process associated with a wide range of conditions, including autoimmune diseases such as SLE, RA, and vasculitis, as well as infections like bacterial sepsis and COVID-19. It also plays a role in cancer progression and metastasis, metabolic diseases like type 2 diabetes and obesity, and organ injuries like pancreatitis and acute lung injury. In this review, we examined and synthesized the clinical evidence supporting the role of NETs in RA.

In RA, neutrophils exhibit an increased propensity to form NETs, thereby increasing exposure to citrullinated auto-epitopes, which are biomarkers of preclinical RA [6, 7]. While studies have associated changes in NET markers with response to treatment, several points remain to be resolved before NET markers can be considered informative in clinical practice.

One challenge lies in achieving consistency in the choice of sample matrix for the quantification of NETs. While analysis of synovial fluid and synovial tissue would best reflect the local inflammatory environment in RA, most investigators report their findings using serum and less commonly, plasma samples [5]. Discrepancies observed between these matrices may arise from differences in sample preparation and storage. Another challenge is the short half-life of NETs and histones in peripheral blood. One study examining plasma histone levels in sepsis reported a histone half-life of just 4.6 min in plasma [70]. In addition, the extracellular DNA scaffolds of NETs are rapidly degraded by circulating DNases and nucleosomes are continuously cleared by the liver, further contributing to their short half-life in circulation [71]. No study has systematically quantified NETs in both peripheral blood and disease-relevant compartments within the same individuals.

**Figure 2:**
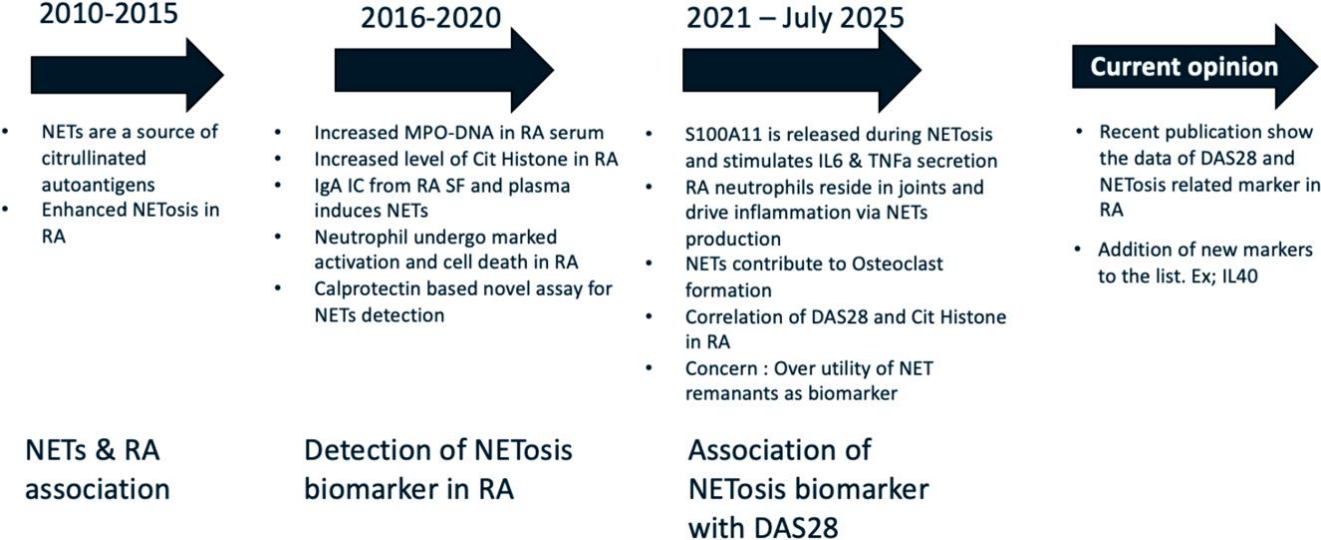
Publication trends over time in the NET and RA field reflect a shift from early studies highlighting NETosis-primed neutrophils in RA to more recent investigations focusing on NET-associated biomarkers and their relevance to disease activity [Created in BioRender. Kumari, S. (2026). https://BioRender.com/fid2w6s].

There is also disparity in the NET markers reported, with some groups favouring individual markers such as citrullinated histones, cell-free DNA, MPO–DNA complexes, or NE, while others adopt a more comprehensive assessment of multiple markers in parallel. It is also important to assess the potential interference of ACPA and RF autoantibodies in NET immunoassays selected. Based on current understanding of NET formation in inflammatory disease states, quantifying markers such as CitH3 alongside MPO–DNA or NE–DNA complexes represents the most reliable and specific approach for detecting NETs [7, 72].

Evidence of a pathogenic role for NETs in RA derives from the association of NETs with disease activity and response to treatment. NET burden decreases in RA patients who respond well to bDMARDs targeting IL-6 (e.g. TCZ) or TNFα (e.g. IFX and etanercept). These treatments not only reduce clinical symptoms and systemic inflammation but also reduce neutrophil activation and NET-forming capacity. For example, treatment with TCZ led to reduced levels of circulating NET components such as cell-free DNA, NE, and MPO, and even suppressed NET formation *ex vivo* [21].

While treatment of RA patients with bDMARDs results in reduction of NET markers, the question remains if NETs are a consequence of the inflammation or an upstream regulator that can influence disease progression. Persistently elevated NET markers in patients with poor treatment responses suggest that NETs may represent viable therapeutic targets.

While correlations exist, causal evidence in humans is lacking, particularly from studies linking NET levels longitudinally with disease progression and radiographic damage. There is a need for longitudinal, compartmentalized studies measuring NETs alongside imaging and immunologic endpoints.

Novel therapeutics targeting NETs have yet to progress to clinical studies evaluating patient outcomes. The most advanced therapeutic approaches targeting NETs in RA include a citrullinated histone H2A and histone H4 targeting antibody CIT-013 from Citryll and AstraZeneca’s bispecific antibody targeting PAD2 and PAD4. Inhibitor approaches targeting PAD4 and DNase have yet to progress to clinical development. The transition to human trials must overcome several hurdles: (i) identification of reliable pharmacodynamic markers to confirm NET reduction; (ii) patient stratification based on NET biomarker profiles; and (iii) demonstration of clinical benefit beyond current standard-of-care therapies. If successful, NET-targeting agents could complement or even synergize with cytokine blockers, providing a new axis for disease modification in RA.

The limitations of this review, relate to the comparison of studies on different aspects of NET biology in RA. The literature screening was conducted by a single reviewer, which may have led to accidental omission of some relevant studies.

Interest in the role of NET formation in RA is increasing. Future studies with standardized methodologies for an agreed set of NET markers in the most relevant biological compartment will advance the field into an era where NET-targeting therapeutics progress through clinical development.

## Data Availability

Not applicable.

## Permission to reproduce

Graphical abstract created in BioRender. Kumari, S. (2026) https://BioRender.com/sx5nxrq. Figure 1 created in BioRender. Kumari, S. (2026) https://BioRender.com/92p23ae. Figure 2 created in BioRender. Kumari, S. (2026) https://BioRender.com/fid2w6s.

## Abbreviations:

ACPAs: anti-citrullinated protein antibodies
DAS28: disease activity score 28
IF: immunofluorescence
IHC: immunohistochemistry
LDGs: low-density granulocytes
NETs: neutrophil extracellular traps
RA: rheumatoid arthritis
